# Study on Hydrogen Sensitivity of Ziegler–Natta Catalysts with Novel Cycloalkoxy Silane Compounds as External Electron Donor

**DOI:** 10.3390/polym8120433

**Published:** 2016-12-16

**Authors:** Hongming Li, Jing Wang, Lei He, Feng Nan, Fan Wang, Wantai Yang, Mingge Zhang, Tianxu Sun, Qigu Huang, Jianjun Yi

**Affiliations:** 1 State Key Laboratory of Chemical Resource Engineering, Key Laboratory of Carbon Fiber and Functional Polymers, Ministry of Education, the College of Material Science and Engineering, Beijing University of Chemical Technology, Beijing 100029, China; lihongming010@petrochina.com.cn (H.L.); wangjing9917@126.com (J.W.); helei8818@163.com (L.H.); 2015200325@mail.buct.edu.cn (F.N.); wangf2011@lzu.edu.cn (F.W.); yangwt@mail.buct.edu.cn (W.Y.); 2 Petrochemical Research Institute, PetroChina, Beijing 102206, China; zhangmingge@petrochina.com.cn (M.Z.); suntianxu@petrochina.com.cn (T.S.)

**Keywords:** Ziegler–Natta catalyst, external electron donor, isotactic polypropylene, high melt flow rate polypropylene

## Abstract

Two novel cycloalkoxy silane compounds (ED1 and ED2) were synthesized and used as the external electron donors (EEDs) in Ziegler–Natta catalysts with diethyl 2,3-diisopropylsuccinate as internal electron donor. The results indicated that the Ziegler–Natta catalysts using ED1 and ED2 as EEDs had high catalytic activities and good stereoselectivities. The melt flow rate (MFR) and gel permeation chromatography (GPC) results revealed that the obtained polypropylene has higher MFR and lower average molecular weights than the commercial EED cyclohexyl methyl dimethoxysilane. The differential scanning calorimetry (DSC) results indicated that new isospecific active centers formed after the introduction of new external donors. The work implied that the novel EEDs could improve the hydrogen sensitivities of the catalyst system and obtain polymers with high melt flow rate.

## 1. Introduction

Polypropylene plays an important role in life and industry due to its excellent physical properties, such as high stiffness, tensile strength, transparency, and low production cost [1,2,3,4]. Despite the rapid growth of single site catalysts, Ziegler–Natta catalysts still hold the dominant position in the production of polypropylene. Usually, in a typical MgCl_2_ supported catalyst, an internal electron donor (IED) such as di-n-butylphthlate (DNBP) and an external electron donor (EED) such as cyclohexyl methyl dimethoxysilane (CHMDMS) are often employed. It has been reported that the molecular structure and performance of polypropylene can be controlled by IED and EED [5,6,7]. Specifically, an electron donor (ED) is generally used to improve stereoselectivity for propylene polymerization [8,9,10,11,12,13]. Although countless potential organic compounds have been tested as IEDs and EEDs, only a few combinations between IEDs and EEDs were found to give satisfactory results [14,15]. Therefore, novel IED–EED combinations with simple structure are still needed to be studied to obtain Ziegler–Natta catalysts for the production of high isotactic polypropylene.

Besides, processability of the polymer is important in the production of polypropylene. The polypropylene products with high MFR—which are easy to process and helpful to improve manufacturing speed—are appreciated by more and more manufacturers. The production of isotactic polypropylene (iPP) with high melt flow rate (MFR) was schematized in the China manufacturing 2025 plan. So far, polypropylene products with good flowability are mainly produced through two methods: degradation by peroxide [16,17], and direct polymerization using novel catalysts with good hydrogen sensitivities [18,19]. The degradation method is to add a peroxide degradation agent to decrease the molecular weight during the reaction. However, the peroxide compounds often have an unbearable smell. In addition, the added chemical agents can result in polymer degradation to some extent. So, this technique is hardly adopted when high quality is required. Meanwhile, the direct polymerization method is being used to produce high MFR isotactic polypropylene in commercial industries. By using novel catalyst with good hydrogen sensitivity, this method can produce high MFR polypropylene with high quality and low cost. Hydrogen sensitivity—referring to the response of the molecular weight and MFR of the polymer to hydrogen regulation in polymerization—is a very important character of Ziegler–Natta catalysts. Usually, good-hydrogen-sensitivity catalysts can be prepared by adding novel IEDs or EEDs. Takefumi reported that polypropylene with high melt flow rate (MFR ≥ 25 g/10 min) was obtained through (R^1^R^2^N)_m_(R^3^HN)_n_R^4^_p_Si(OR^5^)_q_ as external electron donor based on magnesium chloride, magnesium ethylate, and magnesium powder supported Mg–Ti catalysts [20]. Fina technology company utilized 2,2-diisobutyl-1,3-dimethoxy propane (DIBDP) as EED and Mg–Ti supported catalyst using phthalate as IED to prepare polypropylene with MFR up to 67.5 g/10 min [21].

In the previous research, we synthesized four kinds of alkoxy silane compounds employed as IEDs for ethylene polymerization to obtain broad molecular weight distribution by the heterogeneous Ziegler–Natta catalysts via one-pot strategy [22]. In the present research, two kinds of novel cyclo alkoxy silane compounds were synthesized and employed as EEDs based on the spherical MgCl_2_ supported Ziegler–Natta catalysts. Diethyl 2,3-diisopropylsuccinate was employed as IED, which has good hydrogen sensitivity in propylene polymerization. The polymerization results showed that the high isotacticity polypropylene was prepared using these EEDs. It was also found that the novel EEDs could improve the hydrogen sensitivity of the catalyst system and obtain polymers with high melt flow rate. The effects of EED amount on polymerization behaviors were also discussed. The obtained polypropylene was also characterized by differential scanning calorimetry (DSC) and Fourier transform infrared spectroscopy (FT-IR).

## 2. Materials and Methods

### 2.1. Materials

Tetraethoxysilane, Si(OEt)_3_Cl, cyclopentanol, cyclohexanol, 2-ethylhexanol, anhydrous magnesium chloride, and AlEt_3_ with 1.0 M in hexane were purchased from Acros Organics Agent in Beijing, China. Triethylamine, anhydrous ethanol, alcohol, hydrochloric acid, TiCl_4_, toluene, *n*-heptane, and *n*-hexane were purchased from Beijing Chemicals Company (Beijing, China). Toluene and *n*-hexane were further purified by refluxing over sodium under normal pressure for 48 h prior to use. All kinds of alcohols were treated with activated 5 Å molecular sieves under high purity nitrogen atmosphere for one week before use. Decane was purchased from Beijing Chemical Industry Company Ltd. (Beijing, China). Ethylene and propylene (polymerization grade) from Beijing Guangming Chemical and Engineering Company Ltd. (Beijing, China) was used without further purification. 

### 2.2. Propylene Bulk Polymerization

All polymerization manipulations were carried out in a 5 L autoclave after purging all moisture and oxygen by a high-vacuum pump. The desired amount of cocatalyst AlEt_3_, hexane solution of external electron donor, and catalyst were added into the reactor in order at 30 °C. After that, hydrogen was introduced into the vessel. Then, 2.3 L propylene was added into the vessel at 30 °C. After 5 min of pre-polymerization, the temperature of the system was raised to 70 °C. The polymerization occurred at 70 °C for 1 h. Finally, the reaction was stopped, and the obtained product was dried to constant weight in a vacuum oven at 50 °C. The dry polypropylene was weighed, and the catalytic activity was calculated.

### 2.3. Characterization

^1^H NMR spectra were recorded on a Varian INOVA 600 MHz spectrometer (Varian, Palo Alto, CA, USA) in CDCl_3_ solution at 25 °C, and tetramethylsilane was used as reference. All ^1^H chemical shifts were reported in ppm relative to proton resonance in CDCl_3_ at δ 7.26 ppm. Elemental analyses were performed on a Perkin–Elmer 2400 microanalyzer (Perkin-Elmer, Waltham, MA, USA). Ti content of catalyst was measured by Shimadzu ICPS-5000 (Shimadzu, Kyoto, Japan). About 10 mg of the supported catalyst was completely dissolved with HF and HNO_3_ acid. The solution was diluted with distilled water and used for the ICP-AES analysis. FT-IR spectra were recorded by a Nicolet 5DXC FT-IR spectrograph (Nicolet, Madison, AL, USA). The spectra were obtained at a 40 cm^−1^ resolution, and average data were obtained from at least 32 scans in the standard wave number range from 500 to 4000 cm^−1^. The average molecular weight and molecular weight distribution were measured by PL-GPC200 instrument (Varian, Palo Alto, CA, USA) using standard polyethylene as reference and 1,2,4-trichlorobenzene as solvent at 150 °C. DSC thermograms were recorded with a PA5000-DSC instrument (Perkin-Elmer, Munich, Germany) at a rate of 10 K/min. Melt flow rate was measured through GOETTFERT mi-4 melt indexer (GOETTFERT, Buchen, Germany) with diameter of 2.095 mm and die of 8 mm. The isotacticity index (II) of polypropylene was determined through extraction of the sample by boiling *n*-heptane in a soxhlet extractor for 8 h. The weight percentage of the insoluble portion was taken as the II value.

### 2.4. Preparation of Electron Donors

**Scheme 1:**
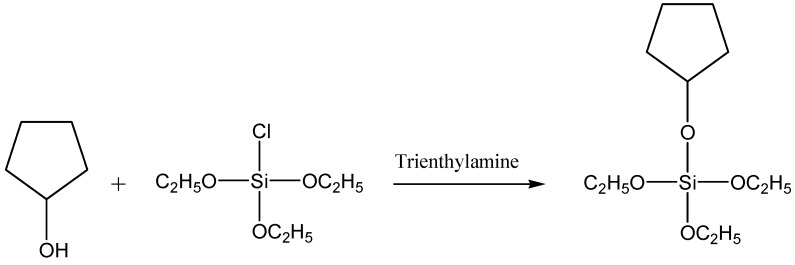
Synthesis of electron donors (ED).

The synthesis of electron donors (ED1 as example) were carried out as Scheme 1.

**Figure 1:**
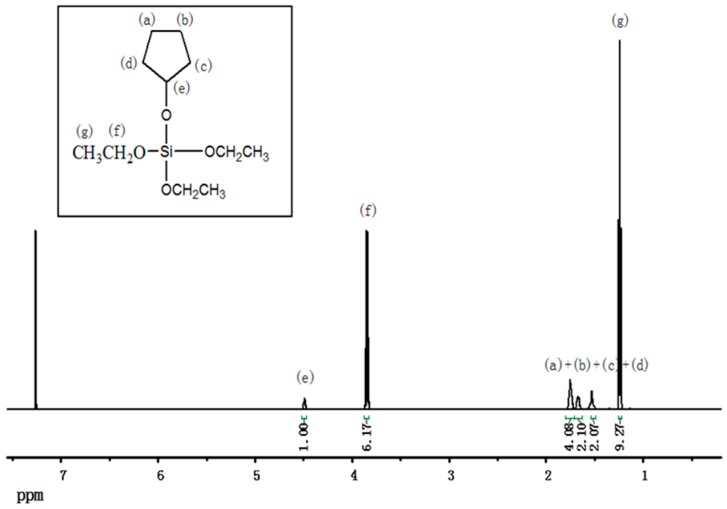
^1^H NMR spectrum of ED1.

Preparation of ED1. Under nitrogen atmosphere, the solution of 1.0 mL cyclopentanol in 100 mL *n*-hexane was added into 300 mL Schlenk flask with a stirring bar. After 1.5 mL triethylamine and 2.2 mL Si(OEt)_3_Cl were dripped slowly into the reactor in order by a syringe at room temperature, the mixture was stirred for 2 h at room temperature. After filtering the mixture, the filtrate was purified by vacuum to get a colorless transparent liquid, triethoxy cyclopentyloxy silane (ED1) with yield of 87.3 wt %. ED1, C_11_H_24_O_4_Si (*M*_W_, 248 g/mol) ^1^H NMR (Figure 1): δ 1.24 (tri, 9H –OCH_2_C**H**_3_); δ 1.51 (m, 2H –CH_2_C**H**_2_CH_2_CH_2_–); δ 1.68 (m, 2H –CH_2_CH_2_C**H**_2_CH_2_–); δ 1.75 (m, 4H –C**H**_2_CH_2_CH_2_C**H**_2_–); δ 3.85 (q, 6H –OC**H**_2_CH_3_); δ 4.98 (m, 1H –C**H**(O)–). E_LEM_. A_NAL_. Calcd: C, 53.23; H, 9.68; O, 25.81. Found: C, 53.21; H, 9.67; O, 25.80.

**Figure 2:**
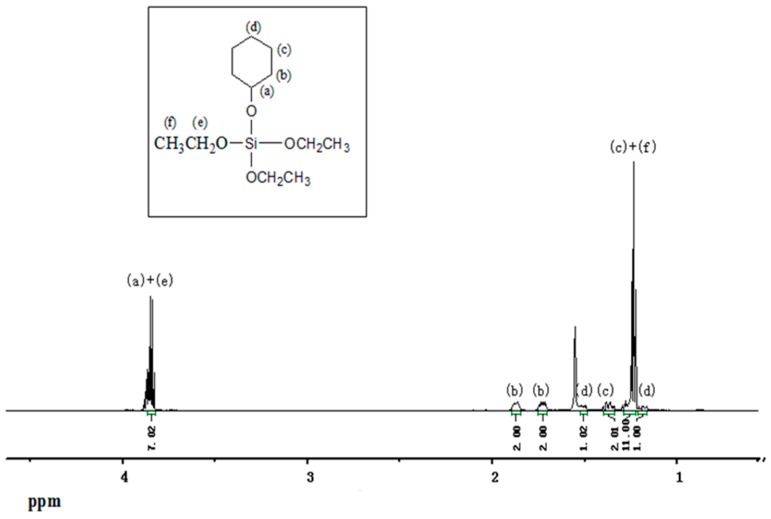
^1^H NMR spectrum of ED2.

Preparation of ED2. Under nitrogen atmosphere, the solution of 1.0 mL cyclohexanol in 100 mL *n*-hexane was added into a 300 mL Schlenk flask with a stirring bar. After 1.3 mL triethylamine and 1.9 mL Si(OEt)_3_Cl were dripped slowly into the reactor in order by a syringe at room temperature, the mixture was stirred for 2 h at room temperature. After filtering the mixture, the filtrate was purified by vacuum to get a colorless transparent liquid, triethoxy cyclohexyloxy silane (ED2) with yield of 90.7 wt %. ED2, C_12_H_26_O_4_Si (*M*_W_, 262 g/mol), ^1^H NMR (Figure 2): δ 1.20 (m, 1H –CH_2_CH_2_C**H**_2_CH_2_CH_2_-CH(O)–); δ 1.24 (tri, 9H –OCH_2_C**H**_3_); δ 1.32–1.40 (m, 4H –CH_2_C**H**_2_CH_2_C**H**_2_CH_2_–CH(O)–); δ 1.51 (m, 1H –CH_2_CH_2_C**H**_2_CH_2_CH_2_–CH(O)–); δ 1.75–1.87 (m, 4H –C**H**_2_CH_2_CH_2_CH_2_C**H**_2_–CH(O)–);δ 3.85 (q, 6H –OC**H**_2_CH_3_); δ 3.81 (m, 1H –C**H**(O)–). E_LEM_. A_NAL_. Calcd: C, 54.96; H, 9.92; O, 24.43. Found: C, 54.95; H, 9.90; O, 24.41.

ED3 is cyclohexyl methyl dimethoxysilane (CHMDMS), which is a mature commercial EED most commonly used in propylene polymerization process. It was prepared according to the patent [23].

### 2.5. Preparation of the Catalysts

MgCl_2_ (1.0 g) and anhydrous ethanol (20 mL) were added into 300 mL Schlenk flask at room temperature with a stirring bar. The mixture was warmed to 80 °C and kept for 2 h, and then transferred into *n*-hexane solution of vacuum grease while hot. After stirring for 20 min, the mixture was filtered, and the residue was washed with *n*-hexane (20 mL × 3). The obtained solid was dried by vacuum to get a white powder spherical support with a yield of 2.45 g. Its component consisted of MgCl_2_·3.5C_2_H_5_OH as determined by micro analysis [24].

Spherical support (1.0 g) was suspended in 50 mL hexane. While stirring, 10 mL TiCl_4_ was dripped into the system at −10 °C. It was slowly warmed to 60 °C, and the desired amount of diethyl 2,3-diisopropylsuccinate was added into the flask. The reaction lasted for 3 h. The obtained residue after filtering was washed with *n*-hexane (20 mL × 3). A brown powder catalyst was obtained with a yield of 0.66 g. The catalyst composition and its principal physical parameters are as follows: Ti content of the catalyst 2.8 wt % determined by ICP-AES, the average diameter of spherical particles 45 μm, the specific surface area ~130 m^2^/g.

## 3. Results and Discussion

The two novel alkoxy silane compounds (ED1 and ED2) and ED3 were used as EEDs for propylene polymerization. Polymerization results are compiled in Table 1. As shown in Table 1, the catalyst systems with ED1 and ED2 as EEDs had high catalytic activities, which were almost the same as ED3. The catalytic activities of catalysts with ED1, ED2, or ED3 as EEDs were lower than that without EED (run 7 and 8). This fact can be attributed to selective positions of EEDs, which suppress reactivity of the aspecific centers and may convert the aspecific centers into isospecific ones [25]. Moreover, with increasing hydrogen concentration, catalyst activities increased in all cases, as chain transfer with hydrogen leads to the regeneration of isospecific propagation [26,27,28].

From Table 1, we can find that the melt flow rates of the obtained iPPs catalyzed by catalysts with novel EED were much larger than expected. When 0.2 MPa hydrogen was added, the MFR of the polypropylene produced by the catalyst with commercial ED3 as EED was 17 g/10 min, whereas the MFR of that with ED1 and ED2 were higher than 120 g/10 min. When ED1 or ED2 were used as EED for propylene polymerization, all the resulted polymers had much higher MFR than ED3. The MFR results suggested that the catalysts with ED1 or ED2 as EED had very good hydrogen sensitivities, which were much better than that with the commercial ED3. This means the novel EEDs could improve the hydrogen sensitivity of the catalyst system and obtain polymers with high melt flow rate. These results affirmed that the hydrogen sensitivity of the catalyst system can be improved by the employment of novel EEDs. Specifically, it is generally believed that there are diversified ways in which donors containing two oxygen atoms coordinate with MgCl_2_ [29]. Different from the traditional EEDs, such as ED3 (CHMDMS), dicyclopentyl dimethoxysilane (DCPDMS), diisobutyl dimethoxysilane (DIBDMS), and diisopropyl dimethoxysilane (DIPDMS) [30], the EEDs synthesized in this work contain four oxygen atoms, which resulted in an accumulation of excess electrons around MgCl_2_ molecules, and thus weakened the coordination ability with TiCl_4_ molecules. The accumulation of excess electrons may increase the odds of 2, 1 insertion, thus leading to the improvement of hydrogen sensitivities [31].

**Figure 3:**
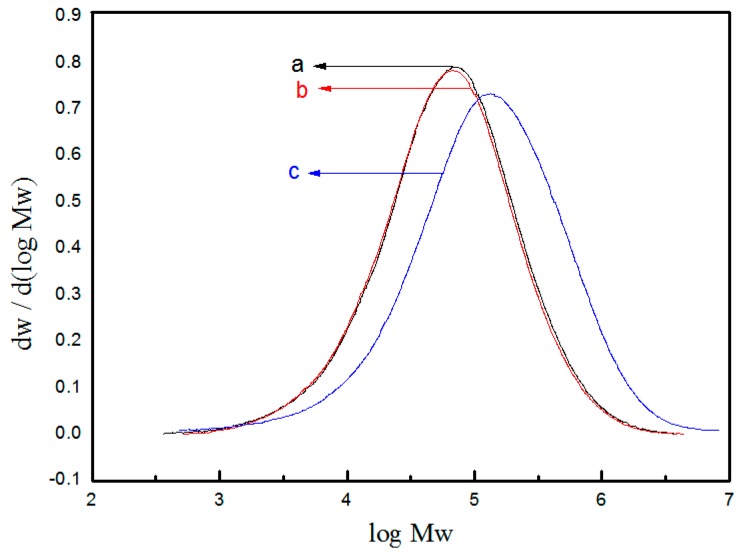
GPC curves of isotactic polypropylene (iPP) (**a** with ED1, **b** with ED2, and **c** with ED3).

Usually, a polymer with high *M*_w_ often has stronger intermolecular interaction, which results in higher viscosity and low MFR [32]. On the other hand, a polymer with high MFR often features low molecular weight or broad molecular weight distribution. The GPC result was presented in Figure 3. The obtained polypropylene catalyzed by catalysts with ED1/ED2 as EED had lower average molecular weight than that with ED3 as EED, implying that these catalyst systems using ED1/ED2 as EED had better hydrogen sensitivities. GPC results agreed with the MFR results. 

The isotacticity of polypropylene was a remarkable characteristic of iPP. The most commonly used catalyst system for iPP in industry contains phthalate as IED and alkoxy silane as EED. ED3 is a typical commercial alkoxy silane EED, most commonly used in the propylene polymerization process. As shown in Table 1, all the isotacticities of iPP catalyzed by the catalyst systems using ED1 and ED2 as EEDs were higher than 97.6%, which were nearly the same as ED3 and higher than that without adding an EED in the catalyst system. This means that the novel alkoxy silane external electron donor has a certain positive role, improving the stability and the selectivity of active centers of the catalyst systems. 

The microstructure of the obtained polypropylene was characterized by FT-IR. Burfield and Loi reported that the isotactic index could be also determined by the following equation [33]:

$$\frac{A_{998}}{A_{973}}=1.08\;\left(\mathrm{II}\right)-0.15,$$

where *A*_998_ is the area of the peak at 998 cm^−1^ in the FT-IR spectra, *A*_973_ is the absorbance area of the peak at 973 cm^−1^, and II is the isotactic index.

The FT-IR results of the resultant iPPs showed that *A*_998_/*A*_973_ ratios were from 0.904 to 0.916, corresponding to the isotactic index from 97.6% to 98.7% (Table 1). The polypropylene catalyzed by the catalyst system without EED exhibited a lower isotactic index of 79.0%. All of the results are consistent with that obtained by soxhlet extraction method. The results confirmed that ED1, ED2, and ED3 as EEDs were effective to improve the stereoselectivity in propylene polymerization.

**Figure 4:**
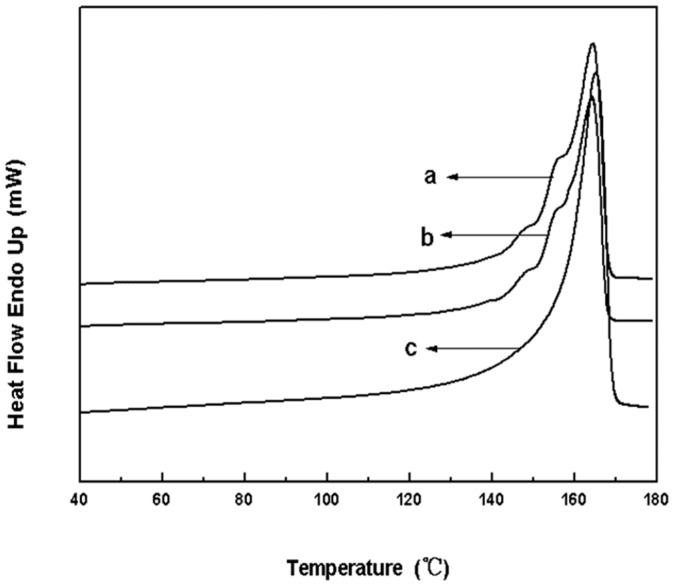
Differential scanning calorimetry (DSC) curves of iPP (**a** with ED1, **b** with ED2, and **c** with ED3).

The titanium-based Ziegler–Natta catalysts are heterogeneous catalysts. They have two types of active centers: isospecific and aspecific. The former produces highly crystalline isotactic polypropylene, and the latter results in amorphous atactic polypropylene. As far as isospecific centers are concerned, they can be divided into several populations of active centers which have different stereospecificity control [34]. Different isospecific centers show different stereoselectivity, chain propagation rate, and chain transfer rate to propylene and Hydrogen. Thus, polypropylene with different isotacticity index, melting point, and molecular weight are produced in polymerization. As a result, the more isospecific centers the catalysts possess, the broader the melting curves of DSC become. From the DSC analysis results shown in Figure 4, we can find that the obtained polypropylene catalyzed by catalyst with ED1/ED2/ED3 as EED all possessed high melting points (curves a, b, and c in Figure 4). This implies that ED1 and ED2—similar to ED3—were favorable for propylene polymerization to form highly isotactic polypropylene. However, the shapes of curves a and b are slightly different from curve c, suggesting that the polymer microstructures are different. The melting curves a and b are broader than curve c. Shoulder peaks in curves a and b are observed, but do not appear in curve c. It is possible that the new isospecific active centers are formed after the introduction of new external donors. Considering that the polymers catalyzed by catalysts with ED1/ED2 as EED had lower average molecular weight and higher MFR than ED3, the newly formed isospecific active center may have higher chain transfer rate to hydrogen. 

The catalytic activity and isotacticity results indicated that the performances of ED1 and ED2 were basically at the same level as ED3, which is a mature commercial EED. This means that ED1 and ED2 are qualified as EEDs. The performance of alkoxy silane as EED is affected by the number of alkoxy groups, size, and nature of the substituent bonded to the silicon atom [35,36]. As far as the EEDs we adopted, the abundant alkoxy groups could block the aspecific sites on the MgCl_2_ layers through competitive complexation and transformation of aspecific into isospecific sites [37,38,39], and even facilitate the formation of new isospecific active sites [40], which contributed to an increase in the stereoregularity of the resultant iPP.

Moreover, the catalysts with ED1 or ED2 as EED exhibited excellent hydrogen sensitivities. When catalysts with good hydrogen sensitivity are used in industrial units, the molecular weight of the polymer can be adjusted by hydrogen in a larger range. This is very meaningful to industrial production: the hydrogen consumption can be reduced, the products can be processed more easily, the manufacturing speed of products can be increased, and more new high-performance polyolefin products can be produced.

## 4. Conclusions

In this research, two kinds of novel cyclol-based alkoxy silane compounds were synthesized and used as external electron donor (EED) to investigate propylene polymerization based on a MgCl_2_/TiCl_4_/diethyl 2,3-diisopropylsuccinate catalyst system. The results confirmed that the catalytic activities and stereoselectivities of catalyst systems with ED1 and ED2 as EEDs were as good as the catalyst with commercial ED3 as EED. The work implied that the novel EEDs could improve the hydrogen sensitivities of the catalyst system and obtain polymers with high melt flow rate. In the near future, the two EEDs will be applied in a pilot polymerization plant of 75 kg/h to verify the effectiveness of improving hydrogen sensitivities, then the two novel EEDs are hopeful to be used in the polypropylene industry. With the market demand growth of high melt flow rate polypropylene, the two EEDs may play an important role for the development of high performance polypropylene.

## Figures and Tables

**Table 1:** Results of propylene polymerization catalyzed by TiCl_4_/IED/MgCl_2_/EED catalyst system.

Run	EED	H_2_ (MPa)	A ^a^ ×10^−3^	II ^b^ (%)	*A*_998_/*A*_973_	II ^c^ (%)	MFR ^d^ (g/10 min)
1	ED1	0.1	10.4	98.6	0.916	98.7	25
2		0.2	12.5	98.3	0.911	98.2	120
3	ED2	0.1	10.9	97.6	0.908	98.0	29
4		0.2	12.7	98.2	0.904	97.6	121
5	ED3	0.1	10.1	97.8	0.909	98.1	7
6		0.2	12.2	98.3	0.907	97.9	12
7	—	0.1	12.4	81.1	0.727	81.2	9
8		0.2	13.8	78.7	0.703	79.0	17

Polymerization conditions: catalyst, Ti content 2.8 wt %, 20 mg; propylene, 2.3 L; temperature, 70 °C; time, 1 h; *n*(Al)/*n*(Ti) = 500:1. ^a^ the catalytic activity, (g PP)·(g cat)^−1^·h^−1^; ^b^ isotactic index calculated by extraction of the sample with boiling *n*-heptane in a soxhlet extractor; ^c^ isotactic index calculated by Fourier transform infrared spectroscopy (FT-IR); ^d^ melt flow rate determined by Melt indexer at 2.16 kg and 230 °C. EED: external electron donor.
